# Editorial: Peanut genomics and biotechnology in breeding applications

**DOI:** 10.3389/fpls.2023.1226637

**Published:** 2023-06-07

**Authors:** Weijian Zhuang, Rajeev K. Varshney

**Affiliations:** ^1^ Center of Legume Plant Genetics and Systems Biology, College of Agriculture, Oil Crops Research Institute, Fujian Agriculture and Forestry University (FAFU), Fuzhou, China; ^2^ WA State Agricultural Biotechnology Centre, Crop Research Innovation Centre, Food Futures Institute, Murdoch University, Murdoch, WA, Australia

**Keywords:** QTL mapping, genomic, food safely, molecular marker, biotechnology

Cultivated peanut (*Arachis hypogaea* L.) is a significant food and oil legume crop cultivated in tropical and subtropical regions globally. Climate change-mediated biotic and abiotic stresses significantly impact peanut productivity and agronomic traits. However, bioinformatics and next-generation sequencing technologies have facilitated the feasible application of peanut genomics and genetics resources in sustainable breeding programs. Genomics- and biotechnology-assisted breeding holds great potential to fast-track the rate of genetic improvement, and design improved peanut cultivars with high yield and quality to ensure food safety. Therefore, this Research Topic on “*Peanut genomics and biotechnology in breeding applications*” presents ten articles from leading experts in this field. This editorial highlights key advances reported in our Research Topic.

Genome-wide identification and characterization of new gene families in cultivated peanut helps to uncover their gene structures, evolutionary relationships, protein interactions, *cis*-elements, expression levels in different tissues and against various stresses, putative miRNAs, and their putative functions using numerous computational tools. In this context, Wang et al. identified 196 *R2R3-MYB* genes in peanut genome, arranged into 48 sub-groups. Of these, 90 genes displayed higher expression patterns against waterlogging stress. Association analysis identified a single nucleotide polymorphism (SNP) located in the third exon region of *AdMYB03-18* (*AhMYB033*), and the three haplotypes of the SNP were highly connected with total branch number, pod length, and root-shoot ratio, respectively, indicating that *AhMYB033* could help to improve the peanut yield. Likewise, Zhang et al. identified 22, 22 and 46 phospholipase Ds (*PLDs*) in *A. duranensis*, *A. ipaensis* and *A. hypogaea*, respectively, and classified them into α, β, γ, δ, ϵ, ζ and φ isoforms. AhPLDs interact with key proteins in lipid metabolic pathways, while ahy-miR3510, ahy-miR3513-3p, and ahy-miR3516 may act as hub regulators. qRT-PCR-based expression analysis indicated that mainly, *AhPLDα3A*, *AhPLDα5A*, *AhPLDβ1A*, *AhPLDβ2A* and *AhPLDδ4A* were highly up-regulated under abiotic stresses.

Wu et al. identified 178 plant protein phosphatase 2C (*PP2C*) genes in cultivated peanut, distributed across 20 chromosomes. Twenty two miRNAs from 14 diverse families targeted 57 *AhPP2Cs*. Some *AhPPC2Cs* were highly expressed in different tissues, while *AhPP2C45* and *AhPP2C134* were highly up-regulated under salinity stress, revealing their role in salinity tolerance in peanut. Another study by Cai et al. identified 116 o-methyltransferases (*OMTs*) genes, divided into three main classes. Twelve miRNAs from various families targeted 35 *AhOMTs* genes. Mostly AhOMTs were up-regulated in various tissues and under phytohormones, drought, and temperature stress. In the future, these novel genes could be genetically engineered to improve peanut yield, quality and stress tolerance.

Genome-wide association study by Oteng-Frimpong et al. discovered 97 SNPs and 17 candidate genes associated with early leaf spot (ELS) and late leaf spot (LLS) diseases in peanut. Of these, 29 unique SNPs were revealed for one or more traits across 16 chromosomes, explaining 0.01-62.76% phenotypic variation. These outcomes offer insights into the genetic structure of ELS and LLS diseases in African peanut germplasm. The identified SNPs and anticipated candidate genes could aid in breeding diseases resistant peanut varieties. Using whole genome resequencing method, Zhang et al. discovered a major quantitative trait loci (QTL, *qRGRB09*) associated with cold tolerance on chromosome B09 (between 46.74 cM-61.75 cM) that is confirmed by KASP markers. A regional QTL mapping assessment confirmed that *qRGRB09* was between the KASP markers, G22096 and G220967 (chrB09:155637831–155854093), and this region contained a total of 15 annotated genes, suggesting their vital role in cold tolerance in peanut. By performing transcriptome and bisulfite sequencing, Liu et al. exhibited that variations in DNA methylation between wild and cultivated peanuts can alter oil content by affecting the expression of peroxisomal acyl transporter protein (*Araip.H6S1B*). In short, they concluded that DNA methylation might act as a negative regulator of lipid metabolic genes and transcription factors, leading to elusive differences in oil accumulation between wild and cultivated peanuts.

After different transgenic screening assays, Huai et al. reported that the red fluorescence protein (DsRed2) could be potentially used as a visual reporter to attain the highest screening productivity and precision in peanut genetic transformation. In another study, Zhuang et al. functionally characterized a novel *Aspergillus flavus* inducible gene (*AhOMT1*) promoter from peanut. In transgenic *Arabidopsis*, *AhOMT1*-promoter showed highly inducible activities under *A. flavus* infection, presenting a new hope for future controlling of aflatoxins contamination through the induction of peanut resistance genes; thus, satisfying the safety concerns of the transgenes. These studies suggest that genetic engineering (gene editing or transgenic breeding) approaches must be fully exploited to improve important agronomic traits and stress tolerance in peanut.

In a review article, Huang et al. presented the scope of different omics approaches, including genomics, transcriptomics, proteomics, metabolomics, miRNAomics, epigenomics and phenomics, for identifying various biotic stress-related genes, proteins, metabolites, metabolic pathways and their networks. Omics understanding is important for developing biotic stress-resistant peanut cultivars to meet the peanut-food demands of the growing world population. The data obtained from these omics approaches can be further exploited *via* genetic or metabolic engineering to design improved peanut cultivars.

In short, this Research Topic contains a plethora of valuable insights into different areas of peanut genetics, genomics, biotechnology, and molecular biology. To further fast-track the development of modern genotypes that can meet future peanut-food necessities and withstand climate change, it is vital to constantly advance genomics and biotechnological techniques, as well as associated statistical methodologies, to help peanut breeders. With the recent advances in genome sequencing technologies, we are anticipated that the peanut community will join hands and develop peanut pan-genome and super pan-genome to further facilitate peanut research and breeding. The integration of different genomics, genetics, and biotechnology tools will be able to guide the peanut community to enhance the peanut production, quality and stress tolerance to meet the food and market demands.

## Author contributions

WZ and RV prepared, reviewed and edited the draft of the editorial. All authors approved the submitted version.

